# Effects and Properties of Deproteinizing Methods in Dentin: A Comprehensive Narrative Review

**DOI:** 10.3390/jfb17060277

**Published:** 2026-06-02

**Authors:** Madalena Belmar da Costa, Ana Mano Azul, Salvatore Sauro, António H. S. Delgado

**Affiliations:** 1 Egas Moniz Center for Interdisciplinary Research (CiiEM), Egas Moniz School of Health and Science, Campus Universitário, Quinta da Granja, 2829-511 Caparica, Portugal; 2 Dental Biomaterials and Minimally Invasive Dentistry, Department of Dentistry, Cardenal Herrera CEU University, CEU Universities, C/Santiago Ramón y Cajal, s/n, Alfara del Patriarca, 46115 Valencia, Spain; 3 Department of Therapeutic Dentistry, I. M. Sechenov First Moscow State Medical University, 119146 Moscow, Russia; 4 Division of Biomaterials and Tissue Engineering, UCL Eastman Dental Institute, University College London, Royal Free Campus, Rowland Hill Street, London NW3 2PF, UK

**Keywords:** bond strength, dentin, deproteinization, endodontic irrigants, enzymes

## Abstract

Dentin deproteinization strategies are being revisited as adjunctive approaches to reduce technique sensitivity, improve monomer infiltration to mineralized dentin, and enhance the longevity of resin–dentin interfaces. This organized narrative review critically summarizes the chemistry, kinetics, biological considerations, and clinical translatability of agents used for smear-layer deproteinization or post-etching deproteinization. Searches of PubMed/MEDLINE, Scopus, and LILACS up to October 2025 were used to identify evidence on oxidizing irrigants (sodium hypochlorite/hypochlorous acid, calcium hypochlorite, peracetic acid, chlorine dioxide), enzymatic proteolysis treatments (bromelain, papain, trypsin/pepsin, and GAG-targeting enzymes), physical approaches (heat and lasers), and post-oxidizer reducing/antioxidant strategies. Oxidizers provide the fastest and most surface-verified organic removal, but their clinical value is limited by concentration- and time-dependent oxidative carry-over, which interferes with free-radical polymerization. Enzymes offer a more selective route, although their support is driven largely by bond strength and morphological outcomes rather than direct surface-chemical confirmation. Heat remains a proof-of-principle method rather than a clinical option, whereas laser protocols are highly parameter-sensitive. Overall, deproteinization should be interpreted through a combined framework of surface chemistry, adhesive compatibility, aging behavior, biosafety, and chairside feasibility. Current evidence supports cautious, protocol-specific development rather than routine clinical adoption, with priority given to clinically realistic time-dose windows and paired surface/aging outcomes.

## 1. Introduction

Deproteinization techniques that alter the surface of dentin for subsequent restorative goals are being increasingly researched. Dentin is composed of approximately 40–45 vol% of a mineral phase of hydroxyapatite crystals (~70 wt%), while the rest of its volume is occupied by 30% organic matrix (20 wt%) and 20–25% water (10 wt%) [1]. The extracellular matrix (ECM) that makes up the organic phase comprises different peptide and polypeptide structures, of which 90% are collagens, with the majority being type I collagen and in a residual 1–3% amount, type III and type V, and including other non-collagenous proteins (10% of the organic matrix) such as phosphorylated proteins [1,2], proteoglycans and glycosaminoglycans. The latter are large and complex water-bound macromolecules claimed to have an especially important role in stabilizing the fibrillar arrangement. Indeed, this latest aspect plays a crucial role in establishing suitable adhesion mechanisms [1,3].

Given the complex, heterogenous and intricate framework seen in dentin, bonding to this substrate is especially difficult. Current bonding techniques rely on a proper interaction between the substrate architecture and the chemical constituents of dental adhesive systems [4]. Ideally, the formation of a stable hybrid layer—a three-dimensional interdiffusion zone, where the polymeric materials should completely interact, envelop, secure and impermeabilize the collagen network that remains after mild or superficial etching [5]—represent the real key of success in operative dentistry. The hybrid layer is coined as the single most important characteristic to achieve successful bonding to dentin in modern operative dentistry. Current interface-engineering studies increasingly treat the hybrid layer not as a passive resin-infiltrated collagen zone but as a coupled chemical–mechanical interface in which factors such as the demineralization depth, volume of exposed collagen, residual water, potential for endogenous enzymatic activity and bacterial biofilm accumulation should be addressed simultaneously [6,7,8,9]. However, managing these processes is highly technique-sensitive [10,11], operator-dependent [10,12] and, most importantly, inherently unpredictable. In addition, the degradation of the exposed dentin ECM is inevitable in time, regardless of the adhesive technique employed [13]. Recent approaches to resin–dentin interface preservation have therefore diverged into at least two mechanistic directions. The first is stabilization of the exposed organic matrix, which has been the focus of attention of a great part of the research in adhesive dentistry in the past 20 years. The other is investigation of the removal of selected organic components, to reduce water retention and proteolytic vulnerability.

In the past decades, different deproteinizing techniques have been studied to deplete and manage the organic phase of dentin. These can be categorized according to their application mode:

(a) Deproteinization before etching coronal dentin when used as a substrate for restorative care, a technique also called smear-layer deproteinization, as reported in the literature since 2001 [14];
(b) Deproteinization of acid-etched dentin—collagen depletion or “reverse hybrid layer” creation, first tested and reported by Gwinnett in 1994 [15].

In the smear-layer deproteinization process, pretreatment of the dentin surface typically involves the application of sodium hypochlorite (NaOCl) or hypochlorous acid (HOCl) prior to etching [16]. The smear layer is a 1–2 μm-thick structure composed of organic components such as collagen fragments, non-collagenous proteins (e.g., dentin phosphoproteins, proteoglycans, and glycosaminoglycans), cellular debris and, in some cases, bacterial byproducts [17]. These organic constituents can hinder adhesive penetration. The deproteinizing agents described above effectively dissolve the organic components within the smear layer, thereby preparing the surface for subsequent adhesive procedures.

Conversely, post-etching deproteinization aims to reduce the exposed organic matrix after acid etching, thereby increasing the relative mineral contribution at the bonding surface [13,15]. This approach has sometimes been described as collagen depletion or creation of a “reverse hybrid layer”; however, the target is not to eliminate collagen alone but the exposed dentin organic scaffold, including collagen, non-collagenous proteins, proteoglycans, and glycosaminoglycans. The intended outcome is a more enamel-like, mineral-rich interface with less water retention and reduced susceptibility to endogenous enzymatic degradation, hydrolysis, and exogenous bacterial/protease-mediated breakdown [18,19]. Nevertheless, the biological and adhesive consequences of this strategy remain protocol-dependent, because excessive or poorly neutralized organic-matrix removal can compromise resin polymerization, dentin mechanics, or pulpal safety.

Despite decades of experimental work, dentin deproteinization remains difficult to translate clinically because the literature is fragmented across different substrates, agent classes, concentrations, application times, adhesive systems, aging models, and outcome measures. Oxidizing agents can rapidly remove organic residues but may compromise free-radical polymerization unless residual oxidants are sufficiently rinsed or neutralized; enzymatic agents may offer more selective proteolysis but have less direct surface-analytical confirmation; and physical strategies such as heat or laser irradiation remain strongly parameter-dependent [20,21,22,23]. Therefore, a clinically useful synthesis must move beyond listing agents and instead compare mechanisms, substrate effects, protocol windows, bond durability, biological safety, and feasibility. Thus, this comprehensive review critically summarizes dentin deproteinization strategies for adhesive dentistry, distinguishing smear-layer deproteinization from post-etching deproteinization and identifying the conditions under which each approach may, or may not, be clinically translatable. Reviewing the effects of these agents on dentin surfaces may not only inform practice but also highlight areas for future research, paving the way for advancements in adhesive dentistry.

## 2. Methods

### 2.1. Review Design

This article was designed as comprehensive narrative review rather than a systematic or scoping review. Its aim was not to answer a narrowly framed intervention question, estimate pooled effects, or map all evidence using PRISMA methodology, but to integrate mechanistic, laboratory, biological, and clinically oriented evidence across heterogeneous available dentin deproteinization protocols. To improve transparency and reproducibility, the review was structured according to principles recommended for high-quality narrative reviews, including clear justification of relevance, explicit aims, a reproducible literature-search description, balanced referencing, consideration of evidence level, and clinically meaningful endpoint reporting [24].

### 2.2. Literature Search

Searches were performed in PubMed/MEDLINE, Scopus, and LILACS from database inception to 15 October 2025, without restrictions on language or year of publication. Searches combined three main blocks: dentin-substrate terms; deproteinization, organic-dissolution, denaturation, irrigant, enzymatic, antioxidant, laser, and thermal-treatment terms; and adhesive-interface or bonding-outcome terms. The core search structure was as follows: (dentin* OR dentinal) AND (deproteini* OR deproteini?ation OR “collagen depletion” OR “collagen-depleted” OR “collagen removal” OR “collagen dissolution” OR “collagen degradation” OR “organic matrix removal” OR “organic phase removal” OR “organic content removal” OR “organic matter removal” OR “organic dissolution” OR “tissue dissolution” OR proteolys* OR proteolytic OR denatur* OR “reverse hybrid layer” OR “smear layer deproteinization” OR “smear layer removal” OR irrigant* OR irrigat* OR hypochlorite OR “sodium hypochlorite” OR NaOCl OR “hypochlorous acid” OR HOCl OR “calcium hypochlorite” OR “peracetic acid” OR PAA OR “chlorine dioxide” OR ClO_2_ OR ClO_2_ OR bromelain OR papain OR trypsin OR pepsin OR hyaluronidase OR chondroitinase OR proteoglycan* OR glycosaminoglycan* OR GAG OR GAGs OR antioxidant* OR ascorb* OR sulfinate* OR “N-acetylcysteine” OR proanthocyanidin* OR EGCG OR laser* OR “Er:YAG” OR “Er,Cr:YSGG” OR “Nd:YAG” OR heat OR thermal OR Carisolv OR Papacarie OR Papacárie OR “Brix 300”) AND (adhesiv* OR bond* OR bonding OR “bond strength” OR “bond durability” OR “bonding performance” OR “adhesion strength” OR “dentin bonding” OR “resin-dentin” OR “resin dentin” OR “resin-dentin interface” OR “dentin-bonding interface” OR “adhesive interface” OR “bonded interface” OR “hybrid layer” OR “hybrid zone” OR “interdiffusion zone” OR “resin tag*” OR “smear layer” OR “smear plug*” OR “etched dentin” OR “acid-etched dentin” OR nanoleakage OR microleakage OR “marginal leakage” OR “marginal adaptation” OR “interfacial adaptation” OR “microtensile bond strength” OR μTBS OR uTBS OR mTBS OR “shear bond strength” OR SBS OR “microshear bond strength” OR “micro-shear bond strength” OR μSBS OR uSBS OR mSBS OR “push-out bond strength” OR “push out bond strength” OR “pushout bond strength” OR “tensile bond strength”). The syntax was adapted to each database. Reference lists of relevant reviews and included papers were hand-searched to identify additional mechanistic, laboratory, biological, and clinical studies.

### 2.3. Eligibility, Evidence Selection and Data Extraction

Eligible sources included in vitro, ex vivo, in situ, and clinical studies evaluating dentin deproteinization or dentin organic-matrix modification in relation to surface chemistry, morphology, polymerization compatibility, bond strength, nanoleakage, hybrid-layer stability, aging, biological safety, or clinical performance. Reviews and systematic reviews were used to contextualize evidence consistency and identify gaps, but primary studies were prioritized when discussing mechanisms and protocol-specific outcomes. Studies focused exclusively on enamel, caries removal without adhesive relevance, or endodontic disinfection without implications for bonding were excluded unless they informed agent chemistry, cytotoxicity, dentin substrate effects, or biological safety. Information was extracted narratively according to agent class, application mode, substrate condition, concentration, application time, mechanism of action, surface-analytical evidence, adhesive-system compatibility, immediate bond strength, aging or nanoleakage outcomes, biological considerations, and clinical feasibility.

## 3. Rationale for Dentin Deproteinization

The process of bur-cutting mineralized tooth tissues during cavity preparation produces a layer of debris that is part mineral and part ECM components (which includes denatured collagen). This smear layer differs depending on the type of bur used in the process—whether diamond or carbide [25]—on the speed and shear stress involved [26], and also on whether it is enamel or dentin [26]. It is poorly adherent and loosely bonded to the underlying dentin, which can compromise adhesive performance, and is thus the subject of research. Despite extensive research on the dentin smear layer, the precise quantification of its compositional elements, particularly the weight/volume percentages of the organic and inorganic components, remains unclear. Studies describe the smear layer as a mixture of organic components, including denatured collagen, cells, blood and saliva contaminants, and inorganic debris like hydroxyapatite, but exact proportions are not reported. Cox (1990) describes the smear layer as consisting of mineralized collagen fibers within an amorphous matrix [27]. The smear layer includes outer amorphous layers, and deeper smear plugs that obturate the dentinal tubules. Similarly, Wang and Spencer chemically confirm with micro-Raman an inorganic/organic matrix in the smear layer [25,28], but the existing research so far has not specifically analyzed the relative percentages of its composition. It is known that dentin permeability can be reduced to values that go up to 86% due to smear plug deposition [29]. Montouris et al. (2004) [30] described, on smear-layer-covered dentin treated with NaOCl, that deproteinization advanced rapidly within 10 s, plateaued by 30–60 s, and peaked at 120 s. This process increased tubule diameter, intertubular porosity, and surface roughness while reducing the intertubular dentin area.

Separate acid etching on the smear layer, using an etch-and-rinse approach with typical orthophosphoric acid, does not completely remove the smear layer, contrary to common opinion, but in turn denatures the collagen and helps form a gelatinous matrix with the mineral content trapped within it [25]. Denaturation evidence of phosphoric acid-etched dentin has also been confirmed with recent ATR-FTIR spectral data and SEM analyses [31,32]. By limiting the acid etching step to 15 s, which is the current recommended time in dentin, the exposed extracellular matrix is preserved [33]. Demineralization of the underlying dentin reveals the collagen network structure and induces the removal of the peritubular matrix [34]. Further complex interactions in the structure are revealed. Proteoglycans (PGs) and glycosaminoglycans (GAGs) form a complex, supramolecular, nanostructured scaffold that maintains the integrity of the collagenous network in dentin [35]. Upon etching, this framework is also exposed. PGs in the peritubular dentinal matrix are resistant to phosphoric acid etching gel [36] and remain within the structure. The same happens with GAGs, which cannot be removed with regular acid etching treatments during bonding procedures [37]. Recent high-resolution TEM clarifies a nanoscale architecture in dentin composed by a woven entanglement of type-I collagen fibrils and hydroxyapatite (HAP) that becomes more isotropic toward the sub-dentin-enamel junction, S-shaped assemblies of elongated HAP crystals wrapping adjacent fibrils, and a transition zone of peritubular/intertubular dentin (~hundreds of nm) where mineralized fibrils run parallel to the tubule axis [38]. This multiscale picture strengthens the rationale for a post-etch deproteinization that removes denatured collagen and PG/GAG hydrogel while preserving apatite continuity, thereby favoring functional-monomer interactions and reducing interfacial water without aggressive chelation. Consequently, post-etching deproteinization aims to deplete this protein content to improve bonding outcomes, while the challenge is to use effective deproteinizing agents in a clinically relevant time [39].

**Figure 1:**
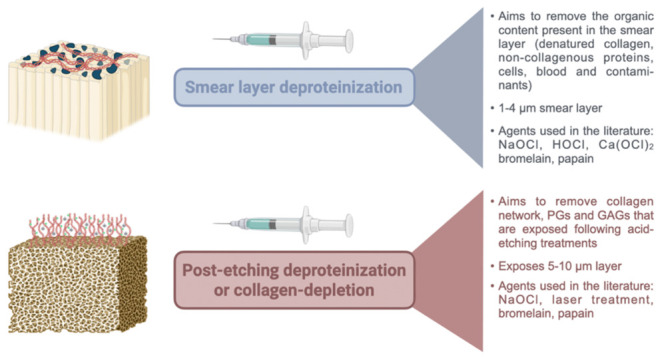
A summary of the two main methods of deproteinization to improve bonding of resin-based materials to dentin; top—smear-layer deproteinization is performed on intact mineralized dentin after cavity preparations; bottom—post-etching deproteinization is performed on demineralized dentin during the bonding procedure.

Although much of the historical literature refers to post-etching organic-matrix removal as “collagen depletion”, this review uses the term “post-etching deproteinization” throughout because the clinically relevant target is broader than collagen alone. The preferred terminology also distinguishes this approach from smear-layer deproteinization, which is performed on mineralized, smear-covered dentin before etching or self-etch adhesive application. The term “collagen depletion” is retained only when referring to terminology used in earlier studies. These two application modes are summarized in Figure 1.

## 4. Chemical Treatments

### 4.1. Irrigants

#### 4.1.1. Sodium Hypochlorite (NaOCl)

NaOCl is widely regarded as the most effective irrigant for root canal treatments due to its excellent antimicrobial properties and tissue-dissolving capabilities [40,41]. With a stable pH of ~11 and availability in concentrations ranging from 1 to 15%, it has become a cornerstone of endodontic therapy [40,42,43]. By leveraging these properties, researchers have explored the potential of this irrigant to address challenges in adhesive dentistry. Its non-specific proteolytic action enables fragmentation of long peptide chains, leading to the non-selective degradation of the organic matrix of dentin [42,44].

The major problem with NaOCl is that it is an oxidizing agent and as such interferes with the polymerization reaction of subsequent light-cured resin-based materials. In this case, premature chain termination occurs because the reactive residual free radicals produced by the oxidation process compete with the propagating vinyl free radicals formed during light-curing. Thus, it is important to be mindful that longer application times of oxidizing irrigants can compromise bonding due to this deleterious effect on the surface of dentin [45].

(i) Effect on smear layer and mineralized dentin

The literature has long-reported on NaOCl being used, in combination with other irrigants such as ethylenediaminetetraacetic acid (EDTA), for the removal of the smear layer. When applied to the dentinal substrate in concentrations of 6–10%, the proteolytic effect of NaOCl induces dentinal tubule opening and creates a porous, irregular collagen structure. This facilitates both the mechanical retention of adhesive monomers and their deeper penetration. However, NaOCl is not highly effective in this mineralized substrate when used in a passive irrigation technique. More recent data found that ultrasonic irrigation methods potentiate the smear-layer removal of irrigants such as NaOCl [46,47].

(ii) Effect on demineralized (post-etched) dentin

The application of NaOCl and its effects on post-etching deproteinization are largely time-dependent. The initial application of NaOCl to recently etched dentin first attacks the hydrogel-like layer formed due to the smear-layer removal and presence of non-collagenous proteins and reveals the collagen network [48,49]. This tends to happen in the first 60 s, as reported in the concentration of 6.5% [48]. Marshall et al. (2001), using AFM/nano-indentation assays described that collagen remnants still persist even after 120 s of 6.5% NaOCl application in etched dentin [48]. Sauro et al. (2009) describes that a two-minute application of NaOCl left residual dentin collagen on both intertubular and intratubular surfaces [39]. ESEM analysis revealed remnants of a hydrogel-like layer, with complete removal achieved only after 45 min of application [39]. NaOCl has also been documented to cause important changes in intertubular collagen and intratubular GAGs [50]. NaOCl enhanced the formation and length of resin tags. Sauro and co-workers [51] also showed that conditioning protocols that remove exposed collagen after etching, with EDTA conditioning followed by a brief NaOCl rinse, reduced hybrid-layer degradation on aging and lowered nanoleakage, yielding more stable microtensile bond strength than conventional approaches. This builds on the fact that eliminating denatured collagen and water-holding residues produces a more hydrolytically resilient, mineral-rich interface, even if immediate bond values are not always higher. Additionally, the combination of NaOCl with sulfinic acid sodium salt, which helps reverse the oxidized surface, improved bond strengths on root canal dentin compared to surfaces treated with etching alone [52].

#### 4.1.2. Hypochlorous Acid (HOCl)

Hypochlorous acid (HOCl), like NaOCl, is an oxidizing agent commonly used as an antiseptic and irrigant in endodontics. HOCl has also been explored as a smear-layer deproteinizing agent to enhance the mineral-to-organic ratio at the dentin bonding surface [53]. Typically applied in concentrations of 40–200 ppm, HOCl partially removes the smear layer and promotes very mild demineralization due to its mild pH (6.2) [54]. Compared to NaOCl, HOCl leaves fewer chlorine residues but also generates oxidizing by-products that may hinder resin polymerization. Despite this limitation, HOCl has demonstrated effective deproteinization, rivaling 6% NaOCl and yielding improved bond strengths in recent studies [16,44]. The wash-out time for HOCl can be improved by incorporating metal chlorides such as SrCl2 or ZnCl2 at a concentration of at least 0.1 M [44]. These additives not only neutralize the oxidizing effects of HOCl but also enhance radical polymerization, allowing shorter clinical application times without compromising immediate microtensile bond strength.

(i) Effect on smear layer and mineralized dentin

Deproteinization with HOCl has been employed in recent studies to investigate its potential in smear-layer removal. Concentrations of 100 ppm HOCl were found to indeed increase the bond strength of different simplified adhesive systems to dentin; however, even use in combination with reduction agents was not able to control bond degradation due to aging [18]. Again, pre-treatment with HOCl and its positive effect is highly dependent upon the application time and the oxidizing potential. Studies indicate that even a brief application of 5 s can enhance bond strength, though this duration also marks the threshold for potential negative effects [55]. It has also been researched as a pre-treatment to improve the adhesion to caries-affected dentin, and in the concentrations of 0.95 and 1.91 mM it was able to remove the superficial denatured collagen in the smear layer of the caries-affected substrate, with a more pronounced effect in the higher concentration [56]. While initial results seem promising, further research is needed to evaluate a wider range of HOCl concentrations and application times, although the potential to oxidize the substrate surface remains, similar to NaOCl.

(ii) Effect on demineralized (post-etched) dentin

On substrates such as eroded demineralized dentin and in low concentrations of 50 ppm, HOCl had a negligible effect compared to 6% NaOCl [19]. No other studies that used HOCl post-etching for organic content depletion were found.

#### 4.1.3. Calcium Hypochlorite [Ca(OCl)_2_]

Calcium hypochlorite, also an oxidizing agent, generally used in water purification and industrial sterilization, has recently received attention in the realm of dentin deproteinization [57,58]. Since NaOCl has proven to cluster several disadvantages, calcium hypochlorite [Ca(OCl)_2_] has been proposed as an alternative that could overcome challenges such as excessive oxidation of the substrate and an altered surface. Due to its relative stability and greater chlorine availability (65%), it can be used in higher concentrations, without leading to possible damages of the substrate, compared to NaOCl [59], since the calcium ions act as stabilizers, thus leading to a milder oxidation profile. The more active chlorine also demonstrates a higher antibacterial effect. Also, the presence of calcium instead of sodium may result in a favored hybrid layer, as it promotes the formation of calcium phosphate or apatite-like phases on the dentin surface [57]. In studies regarding endodontic procedures, calcium hypochlorite has proven to be less harmful to the mechanical properties of dentin when compared to NaOCl, without jeopardizing its antimicrobial activity [58]. Calcium hypochlorite can mainly be found in a granulated powder that should be combined with a saline solution. It can also be found in the form of an aqueous solution, already pre-prepared [59].

(i) Effect on smear layer and mineralized dentin

Calcium hypochlorite has been used in erosion-challenged dentin, with significant deproteinization potential at 1 and 2.5% concentrations, having improved the bond strength in comparison to the positive control, NaOCl, when used with Scotchbond™ Universal [60]. Studies that investigated the behavior of this irrigant in root canal treatments have proven that this substance is able to effectively remove bacteria and the smear layer, with minimal erosion [61].

(ii) Effect on demineralized (post-etched) dentin

Although there are very few studies where calcium hypochlorite was tested in post-etched dentin, the results seem promising. The application of Ca(OCl)_2_ after acid demineralization (35%) in concentrations of either 10% or 15% did not show any significant difference on microleakage values when compared to only acid-etching [61], yet the technique seems feasible and reliable for bonding. In fact, post-etching deproteinization with Ca(OCl)_2_ may lead to higher surface energy as well as permeability of the dentin. Furthermore, SEM results show that the chemical composition of the substrate can in fact be altered by the application of calcium hypochlorite, suggesting the appearance of a mineralized hybrid layer [61,62].

#### 4.1.4. Peracetic Acid (PAA)

PAA is a strong oxidizer that can dissolve organic matter and the smear layer. The formulation consists of hydrogen peroxide (H_2_O_2_) and acetic acid (CH_3_COOH) blended in a stabilizing vehicle at balanced proportions [62]. Its principal active component is a stabilized combination of reactive oxygen derived from H_2_O_2_ together with acetic acid. It has been used, in dentistry, at concentrations ranging from 1–2% in a pH between 4.5–6 [62,63]. In an endodontic study, where 1% PAA was used alone, the solution removed the smear layer and improved the penetrability and bond strength of an epoxy resin sealer to root dentin [63]; its effectiveness was similar to a NaOCl/EDTA/NaOCl sequence. Other studies have corroborated its smear-layer-removing ability through FE-SEM observations [64]. However, decomposition of peracetic acid releases free oxygen that can interfere with free-radical polymerization. It may be that this oxygen is in higher concentrations than that generated by NaOCl [65]. There is no consensus that this irrigant has good organic tissue dissolution ability [66], and thus it may not be the preferred option when the choice is warranted.

#### 4.1.5. Chlorine Dioxide (ClO_2_)

Chlorine dioxide (ClO_2_) is a potent oxidizing gas traditionally used to disinfect water supplies and food-processing equipment [67]. In endodontics, it has been explored as a potential smear-layer deproteinizing irrigant because it can dissolve organic tissue and disrupt bacterial cell membranes while leaving fewer chlorinated residues than sodium hypochlorite [68,69]. Chlorine dioxide has been used in concentrations of 0.014% [69], 0.12% [70], 0.3% [67] and 13.8% [71]. The evidence suggests that while ClO_2_ penetrates dentin and can modify its mechanical properties [71], NaOCl remains the most established deproteinizing irrigant because its proteolytic mechanism is well documented, whereas the capacity for protein removal in ClO_2_ is unquantified other than in one study, which admits it as lesser in comparison [69].

#### 4.1.6. Reducing Agents

Oxidizing pretreatments are effective at dissolving organic residues but can leave reactive oxygen/chlorine species on dentin that quench propagating vinyl radicals, depress the degree of conversion, and reduce immediate bond strength [72]. A short, targeted rinse with reducing/antioxidant solutions is therefore used to neutralize these residual oxidants and restore normal polymerization at the interface. The classical proof-of-concept reducing agent is sodium ascorbate/ascorbic acid, which reverses NaOCl-induced impairment of dentin bonding; original demonstrations (and many replications across etch-and-rinse and self-etch systems) showed recovery of bond strength after brief to several-minute applications of 10% solutions [14,73]. A recent systematic review/meta-analysis confirms that ascorbate-based rinses significantly improve bonding to NaOCl-treated dentin across multiple adhesive families, while emphasizing protocol sensitivity, in parameters such as agent concentration, application time, and adhesive chemistry [72].

Beyond ascorbate, aryl-sulfinates (e.g., sodium p-toluenesulfinate) act as reducing co-initiators that both consume residual oxidants and promote radical formation at the interface. Studies report the restoration of bond strength to NaOCl-treated dentin and, in some models, better longevity after aging relative to untreated controls, likely because sulfinates participate in interfacial redox-initiated cure [74,75]. Thiol donors such as N-acetylcysteine have also shown efficacy as fast electron/thiol sources: when applied after NaOCl, NAC improved push-out or adhesion metrics in coronal and radicular dentin models, offering a practical alternative where chairside time must be kept short [76,77]. A complementary group comprises polyphenol antioxidants. These include grape-seed extract (proanthocyanidins), green tea/EGCG, and rosmarinic acid, all of which the 2024 meta-analysis found to recover bond strength on NaOCl-challenged dentin [72]. Mechanisms include radical scavenging plus possible collagen cross-linking [78], though, again, results vary by adhesive and formulation.

When hypochlorous acid is used for smear-layer deproteinization, adding selected metal chlorides (e.g., ZnCl_2_, SrCl_2_) to the HOCl solution has been reported to shorten wash-out and reduce oxidizing carry-over, yielding immediate bond strengths comparable to or better than HOCl alone [53,79]; functionally, this achieves the same goal of restoring polymerization while preserving the cleaning benefit of the oxidizer. It is important to note that not all antioxidants are equally effective, however: for example, sodium thiosulfate shows inconsistent or limited benefit in controlled comparisons, and the heterogeneity of protocols remains a barrier to direct translation [80].

### 4.2. Enzymes

**Figure 2:**
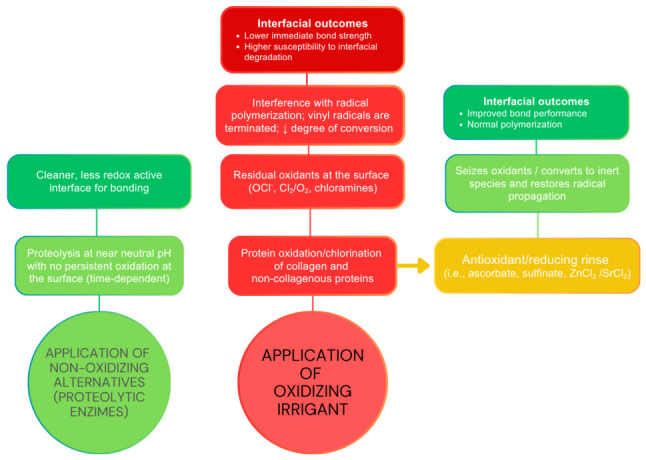
Redox pathway at the dentin–adhesive interface after oxidizing pretreatments. The application of oxidizing irrigants oxidize/chlorinate surface proteins and leave residual oxidants that quench propagating vinyl radicals, depressing the overall degree of conversion and bond strength. A short reducing/antioxidant rinse (ascorbate, sulfinates, selected metal chlorides) scavenges these species and restores radical polymerization. For comparison, the dashed sidetrack illustrates enzyme-based deproteinization (proteolysis at near-neutral pH), which removes organic residues without persistent oxidants, producing a less redox-active interface.

Unlike oxidizers, which cleave peptides indiscriminately and leave an oxidized surface that often quenches free-radical polymerization [81,82], proteolytic enzymes act by substrate-driven catalysis at or near physiologic pH. This targets denatured and exposed peptide domains without generating persistent oxidizing by-products. In practical terms, this means (i) organic removal without mineral loss, (ii) lower risk of polymerization inhibition, and (iii) a cleaner, less redox-active interface for vinyl monomers to cure [83,84]. Enzymes act on organic residues without chelating mineral, so the post-treatment surface retains apatite available for functional monomers (e.g., 10-MDP). These mechanistic differences help explain why immediate bond strengths after enzymatic deproteinization are often maintained or improved without special neutralization steps. To better illustrate these differences, a schematic is shown in Figure 2.

#### 4.2.1. Bromelain

Bromelain is a complex blend of thiol-endopeptidases and other components, extracted from the fruit or stem of pineapple, including phosphatases, glucosidases, peroxidases, cellulases, glycoproteins, and carbohydrates, many of which are not yet fully identified [85]. It also contains several proteinase inhibitors [86]. Bromelain is known to degrade and deplete collagen and other extracellular matrix components [87], since it cleaves the internal peptide bonds found in the protein chains of denatured matrix components [88]. It offers a safer alternative to NaOCl, with good stability, superior biocompatibility, and added anti-inflammatory properties, as tested in endodontic studies [89].

(i) Effect on smear layer and mineralized dentin

When used as a smear-layer deproteinizing agent, at 10% concentration for 30 s, it is effective in increasing tubular diameter and improving the formation of resin tags and the subsequent retention [90]. When tested alongside other novel formulations, such as bromelain-chloramine-T and bromelain-chlorhexidine gel, the effect of bromelain in the smear layer is visible but less significant. In terms of degradation of the smear layer and a higher amount of opened dentinal tubules, chloramine-containing formulas with bromelain presented better results [88].

(ii) Effect on demineralized (post-etched) dentin

When applied after acid etching of dentin, bromelain has shown interesting results in achieving post-etching deproteinization of the exposed organic matrix [91,92]. Unlike NaOCl, bromelain does not appear to compromise the bond strength of adhesive systems applied to dentin and may enhance bonding performance under selected protocols. Dayem et al. (2013) demonstrated that bromelain improved collagen removal, enabling better monomer diffusion, reduced leakage and increased dentinal permeability [92]. The use of 8% bromelain for 1 min, in comparison to 5.25% NaOCl, was found to improve the immediate microtensile bond strength to dentin [93]. Alahdal et al. (2025) found a significant increase in the shear bond strength results, outperforming all other groups, and in resin tag length, for the group treated with 10% bromelain for 30 s, after 35% phosphoric acid for 15 s [94]. On the other hand, Sharafeddin et al. (2024) [95] did not report significance in shear bond strength results when using 6% and 10% bromelain, although the group in which 6% bromelain was used showed higher SBS values. These studies are still insufficient and more are needed to discern the true potential of this enzymatic treatment [94,95].

#### 4.2.2. Papain

Papain, a cysteine proteolytic enzyme extracted from the ripe fruit of Carica papaya, has garnered attention as a deproteinizing agent due to its specificity and safety profile [96,97]. Sharing similarities with human pepsin, papain can effectively remove the organic components of enamel, dentin, or carious lesions without adverse biological effects, thus maintaining intact collagen fibrils. Additionally, its antibacterial and anti-inflammatory properties further enhance its utility in dental applications. Furthermore, it has been reported to enhance bond strength when used in concentrations between 8–10% [98].

(i) Effect on smear layer and mineralized dentin

Papain has been used and tested in terms of removal of carious lesions, supporting a minimally invasive technique. Nowadays, papain is considered an effective chemo-mechanical agent for caries removal, with minimal adverse effects. Studies have shown that papain application can lead to moderate or minimal smear-layer formation [99]. As far as smear-layer removal is considered, studies are mainly focused on endodontic treatment. Even so, it is relevant to underline that 1% papain application is able to remove the organic components of the smear layer inside of the canal. Concerning the effect on collagen, Kusumasari et al. (2021) [100] recently showed that papain can be effective in smear-layer deproteinization, even when compared to NaOCl.

(ii) Effect on demineralized (post-etched) dentin

In a comparative study by Khatib et al. (2020), 8% papain was applied to dentin for one minute, followed by rinsing with distilled water and subsequent adhesive bonding. While papain achieved higher bond strength than NaOCl, bromelain exhibited the highest performance among tested deproteinizing agents [93]. Studies have shown that concentrations of 8–10% papain yield significant improvements in bond strength based on protein depletion, outperforming potentially oxidizing agents that may compromise the polymerization reaction of free-radical vinyl monomers. On the other hand, as shown for bromelain, some studies demonstrated that papain application resulted in no significant differences in terms of shear bond strength, although the group treated with 15% papain demonstrated higher results compared to 10% [95].

#### 4.2.3. Enzymes Targeting PGs and GAGs (Chondroitinase ABC, Hyaluronidase, Pepsin, Trypsin)

Hyaluronidase, as a GAG hydrolase, can target chondroitin and dermatan sulfate and reduce hyaluronic-acid–mediated water retention in etched matrices to aid infiltration [101]. Evidence for dentin is mostly exploratory. Regarding chondroitinase ABC, it is a GAG lyase that selectively degrades chondroitin/dermatan sulfate GAG chains. Studies showed changes in μTBS and altered resin infiltration after C-ABC pretreatment of etched dentin. It is important to note that the protocols often used very long incubations (not yet chairside-ready). Pepsin has been explored experimentally as part of “self-limiting proteolysis” concepts on demineralized dentin; it can be regarded as mechanistically plausible, but acidic pH and long exposures mean it is not a clinical option. Trypsin has used post-etch to remove PGs and some non-collagenous proteins, since reducing the water-binding gel can significantly raise bond strength. This is one of the better-documented enzymatic routes outside papain/bromelain. One of its main disadvantages, however, is that the protocol is in a non-relevant clinical time frame.

#### 4.2.4. Commercial Enzymatic Solutions

Both Papacárie^®^ (F&A Laboratório Farmacêutico, São Paulo, Brazil) and Carisolv^®^ (MediTeam, Sweden) are commercial products designed for minimally invasive carious tissue removal and dentin deproteinization, relying on chemo-mechanical mechanisms to degrade partially disrupted collagen molecules and effectively eliminate infected soft dentin, but also, recently, the smear layer.

Papacárie^®^ is a papain-based gel that acts as a deproteinizing agent, specifically targeting infected dentin. Its primary component, papain enzyme, interacts selectively with degraded collagen in infected dentin, rendering it friable and easily removable with hand instruments [102,103]. The gel also contains chloramines and toluidine blue, which enhance its ability to dissolve the organic structure of the smear layer effectively [104]. The specific proteolytic activity of papain makes it highly effective in smear-layer deproteinization, as confirmed by Kusumasari et al. (2021), where Papacárie^®^ outperformed Carisolv^®^ and low-concentration NaOCl in organic phase removal [100]. A 60 s application of Papacárie^®^ was tested on dentin surfaces and was shown to reduce the gap formation before the application of self-etch adhesives in Swept-Source Optical Coherence Topography (SS-OCT) analysis [105].

Carisolv^®^, conversely, utilizes a two-syringe system: one syringe contains a carboxymethylcellulose-based gel with amino acids (e.g., glutamine, leucine, lysine), and the other contains 0.95% NaOCl [106,107,108], which was increased from 0.5% initially [109]. Unlike higher concentrations of NaOCl used in traditional deproteinization (e.g., 5.25%), Carisolv^®^ relies on this lower concentration in gel form, allowing for a controlled, localized action. The proteolytic activity of the product is primarily attributed to NaOCl, which dissolves the organic phase of the smear layer and prepares the dentin for subsequent adhesive procedures [106]. Both Papácarie^®^ and Carisolv^®^ have been proven to effectively deproteinize dentin, with specific strengths. Papacárie^®^ excels in selective action on infected dentin, thanks to its papain enzyme, while Carisolv^®^ offers controlled and minimally invasive application due to its gel-based NaOCl formulation. In comparative studies using ATR-FTIR analysis, both products showed significant organic phase elimination, with Papacárie^®^ performing similarly to 6% NaOCl but significantly better than Carisolv^®^, likely due to lower NaOCl concentration in Carisolv^®^.

In 2011, a new version of Papacárie^®^ was developed, Papacárie^®^ Duo, featuring a higher viscosity, which leads to less error and higher durability [110]. Moreover, another relevant papain-based product was developed—Brix 300. This agent contains 10% papain and relies on an Encapsulated Buffer Emulsion (EBE) mechanism for greater stability and fixation of the gel, without compromising its pH. This new formula has showed an enhancement of the proteolytic activity, leading to better results in terms of collagen deproteinization. Authors such as Kusumasari et al. (2021) [100] have shown that deproteinization of the smear layer induced by Papacárie^®^ Duo facilitates the removal of damaged collagen fibrils, creating a layer of stable collagen and thus aiding the formation of an optimal chemical bonding to dental adhesives. From SEM observations, little to no smear layer was present when compared to conventional methods. Coelho et al. (2025) [110], when comparing both Papacárie^®^ Duo and Brix 300 to the use of conventional mechanical caries removal with rotatory instruments, did not achieve a significantly higher shear bond strength when these materials were used. This study pointed out that the higher concentration in Brix 300 could lead to a stronger proteolytic activity; nonetheless, the application of Brix 300 did show a severe decrease of bond strength and shear modulus at the interface. Moreover, when compared to NaOCl and chlorine dioxide, Brix 300 did not show promising results in terms of shear bond strength and presented the lowest mean microleakage score [110]. There are no other studies that tested Brix 300 as a deproteinizing agent, and thus additional results are urgent.

#### 4.2.5. Caveats in Enzymatic Treatments—Kinetics

Compared with hypochlorite oxidizers, which show fast, surface-verified deproteinization of smear-covered dentin (with microscopy and spectroscopic endpoints demonstrating tubule opening and organic loss within ~10–120 s), proteolytic enzymes (papain, bromelain) act by catalytic cleavage and generally require longer exposure to reach comparable functional improvements. Current support for short, post-etch windows (≈30–60 s at 8–10%) is predominantly derived from mechanical outcomes, typically bond strength and tag morphology in SEM, rather than surface analytics. Thus, while enzymes can deliver clinically workable improvements without introducing oxidizing by-products that risk polymerization inhibition, it is not yet possible to claim surface-equivalence to oxidizing agents. Until direct surface-level confirmation (e.g., ATR-FTIR/μRaman for Amide-I/III loss, AFM/profilometry for roughness and SEM for surface morphology) is available, enzyme protocols should be presented as selective, lower-redox alternatives whose effectiveness has been demonstrated mainly through bonding outcomes. Kinetics in these agents are more sensitive to concentration, agitation, and substrate condition.

## 5. Physical Treatments

### Heat Treatment

In addition to chemical oxidizers and enzymes, heat offers a purely physical route to remove or denature dentin organic content. Heat treatment can denature and coagulate proteinaceous structures, including collagen and water-retaining PG/GAG gels. Denaturation temperatures vary according to hydration and mineralization state: the thermal decomposition temperature (Td) of mineralized dentin ranges from 160 °C to 186 °C, depending on hydration and age, whereas demineralized dentin shows lower thermal stability unless dehydrated [111]. Heat is therefore mechanistically useful for demonstrating how a protein-depleted surface behaves, but it should not be confused with a clinically applicable deproteinization procedure because the temperatures required would be biologically unacceptable in vivo.

Thus, heat treatment is best interpreted as proof-of-principle evidence that extensive removal or denaturation of the organic mesh is possible while preserving the inorganic phase under controlled laboratory conditions [112]. This shows that total or near-total deproteinization is physically achievable, but the finding should be used to inform the design of gentler chemical or enzymatic routes rather than to support chairside thermal application.

## 6. Physico-Chemical Treatments

### Laser Treatment

Many studies have proven over the decades that most lasers are harmful, leading to negative thermal effects on dentin and pulp. With the discovery of two types of laser—Er:YAG and Er,Cr:YSGG—it was shown that heat treatment can be used in dentinal substrates without biological damage. These lasers, specifically Er:YAG, were initially mainly used for treating carious lesions, as a minimally invasive technique. Nonetheless, more recently, lasers have been the subject of numerous investigations regarding deproteinization abilities [113,114,115,116,117,118]. Different lasers and different parameter treatments have been reported in the literature (Table 1).

Specifically, the use of neodymium-doped yttrium aluminum garnet (Nd:YAG) lasers has been shown to induce significant physical, morphological, and chemical alterations on dentin. The high-energy interaction with the surface of dentin promotes localized melting and subsequent recrystallization, leading to the formation of an irregular topography [119,120]. This process often results in partial evaporation or fusion of the smear layer with the underlying dentin, potentially influencing subsequent adhesive procedures and demineralization dynamics. Dayem et al. (2009) demonstrated that Nd:YAG lasers more effectively remove the collagen network from acid-etched dentin compared to 10% NaOCl [121]. Similarly, Kasraei et al. (2021) [122] found that Nd:YAG and 940 nm diode lasers reduce marginal microleakage in in vitro Class V composite restorations. Resaei-Soufi et al. (2019) further noted that applying the Nd:YAG laser after primer application, or diode laser after applying the bonding agent application, significantly improves microtensile bond strength [123]. Out of all the lasers though, the most documented one is Er:YAG. It has been proven to, apart from other surface changes, generate a smear-layer free bonding layer in dentin [116]. While lasers are known to generate heat that promotes resin penetration, no consensus exists regarding the optimal protocol [123]. Studies that investigated bond strength outcomes indicate increased values when lasers are applied after the use of adhesives [124], whereas others report better results with pre-application [125].

(i) Effect on smear layer and mineralized dentin

Most studies focused their research on Nd:YAG and Er,Cr:YSGG lasers. When acid etching is carried out after Er,Cr:YSGG laser irradiation, the inorganic tissue on dentin is successfully demineralized, and dentinal tubules are opened, leading to a more suitable bonding environment. Moreover, Lee et al. (2007) [126] also demonstrated that while the laser itself can harmfully affect dentin, when followed by acid-etching it can elevate the tensile bond strength. Laser irradiation with Er,Cr:YSGG, in conjunction with NaOCl and EDTA, was able to alter collagen content in laboratory studies [127]. When using the Er:YAG laser before the adhesive procedures, the hybrid layer was not observed, demonstrating a true demineralization [128], whereas a thin hybrid layer could be analyzed when the Nd:YAG laser was used. When referring to mineralized dentin, the mechanism carried out by laser irradiation leads to a rough surface with opened tubules and deprived of a smear layer [129,130]. The heat from laser irradiation can, in fact, eliminate free radicals and modify the dentinal surface, thus providing a suitable bonding substrate. Regarding CO_2_ laser irradiation, it has been noted that it concentrates higher levels of energy in a small area [129], inducing chemical and morphological changes with melted enamel prisms, ultimately increasing the results for microleakage.

(ii) Effect on demineralized (post-etched) dentin

Studies have reported that lasers can modify acid-etched dentin in ways that resemble post-etching deproteinization, but these findings should be interpreted cautiously because laser effects depend strongly on wavelength, energy density, pulse duration, water cooling, dentin depth, and adhesive chemistry. Dayem et al. (2009) [121] reported that treatment of acid-etched dentin with a Nd:YAG laser increased adhesive penetration compared with 10% NaOCl, whereas other studies showed that solvent type and adhesive family influenced the outcome. Recent meta-analytical evidence for Er:YAG pretreatment indicates comparable performance to mechanical preparation in some comparisons but inferiority to conventional etch-and-rinse when used alone, with high heterogeneity and exclusively laboratory-based evidence [131,132]. Therefore, laser-based deproteinization should be presented as parameter-sensitive and experimental rather than as a standardized clinical protocol.

## 7. Biological Effects, Pulp Safety, and Clinical Feasibility

The biological interpretation of dentin deproteinization requires caution because most available evidence comes from laboratory models using extracted teeth, standardized smear layers, and direct surface application under conditions that do not reproduce pulpal pressure, remaining dentin thickness, odontoblast responses, dentinal fluid flow, salivary dilution, or intraoral clearance.

Firstly, NaOCl use should be considered a concentration- and time-dependent intervention rather than a generic option. Its antimicrobial and tissue-dissolution capacity explains its widespread endodontic use [42], but this benefit is chemically linked to collagen degradation within mineralized dentin and the formation of a collagen-sparse mineral matrix with reduced flexural strength [42]. FTIR data have shown that NaOCl-induced dentinal collagen changes can extend at least 0.5 mm from the canal wall [40], while exposure to 5.25% NaOCl significantly increases collagen degradation and reduces the flexural strength of mineralized dentin [43]. These effects are consistent with broader evidence that NaOCl adversely alters dentin mechanical properties [41]. In adhesive protocols, this biological caution overlaps with polymerization concerns. Sodium ascorbate can reverse compromised bonding to oxidized etched dentin [14,73], sulfinates can apparently improve bonding to NaOCl-treated dentin [74,75], and N-acetylcysteine-containing strategies have been associated with improved adhesiveness/biocompatibility in radicular models [76,77], but antioxidant effects remain protocol- and material-dependent [72,80]. Consequently, short exposure, tailored concentration, copious rinsing, and the need for reducing or using antioxidant neutralization should be regarded as part of the safety profile of using NaOCl.

The evidence for alternative oxidizers is more formulation-specific. For peracetic acid, Brandão-Neto et al. compared 2% PAA with 5.25% NaOCl and 2% CHX and found greater cell viability for PAA than for CHX or NaOCl but weaker antimicrobial activity against mature *Enterococcus faecalis* biofilm [62]. Viola et al. further showed that PAA cannot be interpreted as a single biological entity: different PAA formulations varied in smear-layer removal, dentine erosion, cytotoxicity, and antibiofilm activity [64]. Thus, PAA should be discussed as formulation-, concentration-, and indication-dependent rather than as a universally safer oxidizer. For ClO_2_, the current evidence is stronger for antimicrobial activity and dentin modification than for direct pulp-cell safety. Studies have evaluated antimicrobial activity and dentin bond strength [67], endodontic use [68], ATR-FTIR-detectable dentin chemical changes [69], disinfection depth inside dentinal tubules [70], and effects on microhardness and surface roughness [71]. Therefore, ClO_2_ should be presented as a promising but still understudied.

Enzymatic approaches may offer a less redox-active biological profile, but they are not automatically risk-free. A bromelain/chlorhexidine/EDTA irrigant showed better tissue-response behavior than 2.5% NaOCl in a rabbit subcutaneous model, with less overall irritation, less oedema, and earlier healing [89]. This supports bromelain as a biologically attractive alternative to hypochlorite-based organic dissolution, although the tested formulation combined bromelain with chlorhexidine and EDTA and should not be interpreted as evidence for all bromelain protocols [89]. Papain-based systems require the same distinction between purified enzyme and commercial gels. Current manuscript references support papain-related caries removal, formulation differences, deproteinization, and bonding effects [96,97,98,99,100,101,102,103,104,105,106,107,108,109,110], but stronger cytotoxicity claims would require adding direct pulp-cell studies. For example, Papacárie Duo^®^ has been reported to be cytotoxic and to reduce cell viability at 5%, but not at 0.5%, in macrophage and dental pulp-cell cultures [133]; more recently, chemical caries-removal products showed product- and concentration-dependent cytocompatibility in hDPSCs, with IC50 values of 0.596% for Brix 3000^®^, 0.052% for Papacárie Duo^®^, 1.034% for NATURAL-CARE, and 0.020% for Cariesolut [134].

Laser-based protocols require particular caution because their effects are governed by many different parameters (wavelength, energy density, pulse duration, water cooling, tip distance, repetition rate, adhesive strategy, and remaining dentin thickness). The deproteinization literature mainly supports parameter-dependent chemical and morphological modification rather than assessing direct pulp-cell safety [116]. Under simulated pulpal pressure, Nd:YAG laser pretreatment has been shown to alter dentin chemical composition and affect immediate and long-term bond strength [119], while meta-analytic evidence indicates that laser pretreatment does not consistently outperform conventional dentin preparation and that outcomes depend on laser type and adhesive strategy [131,132]. Therefore, even when lasers appear favorable for surface modification, phenomena such as excessive thermal accumulation, melting, and recrystallization should be considered both a biological and adhesive risk [116,131,132]. Heat treatment, in contrast, should be regarded only as a laboratory proof-of-principle for protein removal; the temperatures required to denature or remove the dentin organic matrix are not clinically feasible to preserve pulp vitality [111,112].

## 8. Limitations

This review has limitations inherent to its design. Consequently, the conclusions should be interpreted as a critical, mechanism-oriented descriptive synthesis rather than as pooled evidence or clinical recommendations. The underlying literature is highly heterogeneous with respect to the state of the dentin substrate, its depth, smear-layer standardization, etching protocol, deproteinizing agent, concentration, application time, adhesive system, outcome measure, and aging method. Most studies are in vitro or ex vivo and report immediate bond strength without paired surface chemistry or long-term aging. Clinical evidence remains scarce and is currently dominated by trials of post-etching NaOCl that did not show clear clinical superiority over conventional etch-and-rinse bonding without deproteinization [135,136,137]. These limitations restrict the strength of chairside recommendations and reinforce the need for clinically realistic protocols proven by RCT designs and analogous higher evidence clinical studies.

## 9. Critical Synthesis and Clinical Implications

The comparative properties of the major dentin deproteinization strategies are summarized in Table 2. Across agents, the most consistent finding is that deproteinization cannot be judged by bond strength alone. The same surface intervention may remove organic residues, increase tubule exposure, and alter surface energy but also may inhibit polymerization or change long-term water sorption, depending on its application factors. For NaOCl, meta-analytical and laboratory evidence remains heterogeneous: high-concentration or prolonged exposure can impair bonding if oxidative carry-over is not controlled, whereas reducing or crosslinking agents such as sodium ascorbate, proanthocyanidins, rosmarinic acid, EDTA, or sulfinates may recover bond strength in selected protocols [72,78,128]. For HOCl, the available evidence suggests a potentially wider chairside window than NaOCl in smear-layer deproteinization, but benefits remain dependent on application/wash-out times and the adhesive system used [53,79]. Enzymatic treatments appear attractive because they avoid persistent oxidizing species, yet many short chairside protocols are supported mainly by mechanical outcomes and SEM morphology rather than direct surface-chemical confirmation. Laser protocols add further complexity because apparent improvements can reflect roughness, microretention, smear-layer modification, or thermal alteration rather than selective organic-matrix removal.

Clinical translation remains the weakest part of the evidence base. Only two randomized clinical trials using post-etching 10% NaOCl in non-carious cervical lesions were identified [133,134]. Both reported that deproteinization did not significantly improve clinical performance compared with conventional bonding at three or five years. Importantly, these trials evaluated a specific oxidizing protocol and did not incorporate reducing neutralization, enzymatic deproteinization, or contemporary universal-adhesive optimization; therefore, they should not be interpreted as definitive evidence against all deproteinization strategies. Instead, they show that laboratory improvements in morphology, permeability, or immediate bond strength do not necessarily translate into superior restoration survival when protocol compatibility and long-term degradation are not optimized.

From a clinical standpoint, dentin deproteinization should currently be viewed as an adjunctive and protocol-specific strategy rather than a universal bonding step. Its potential value is greatest when it reduces water-rich organic remnants, improves access of functional monomers to minerals, limits nanoleakage, or stabilizes the interface after aging. However, these theoretical advantages must be balanced against additional chairside time, technique sensitivity, possible polymerization inhibition, uncertain compatibility with simplified or universal adhesives, and limited clinical validation.

The most defensible translational position is therefore conservative: oxidizing protocols require short, controlled exposure and effective rinsing or neutralization whenever they are used. Enzymatic protocols still lack surface-analytical and aging validation. Laser protocols require parameter standardization and pulp-safety confirmation, while heat treatment is generally reserved as a laboratory comparator. Future studies should pair surface chemistry (e.g., ATR-FTIR, micro-Raman, XPS, AFM/SEM) with polymerization metrics, nanoleakage, water aging, thermomechanical cycling and, ultimately, randomized clinical outcomes.

## 10. Conclusions

Dentin deproteinization remains a mechanistically interesting approach for modifying the resin–dentin interface, but the current evidence is still far from recommending its routine incorporation into clinical restorative protocols. Although oxidizing agents, proteolytic enzymes, lasers, and thermal approaches can modify or remove organic components from smear-covered or acid-etched dentin, the available studies are in general in vitro and ex vivo studies. Consequently, favorable immediate bond strength or surface morphology findings cannot yet be interpreted as proof of clinically meaningful improvement in restoration longevity.

At present, the strongest conclusion is that deproteinization is a meaningful research strategy rather than a validated chairside protocol. Oxidizing agents such as NaOCl, HOCl, Ca(OCl)_2_, PAA, and ClO_2_ are chemically effective but remain limited by oxidative carry-over that induces polymerization interference and biological safety concerns. Enzymatic approaches such as bromelain and papain may be more selective and less redox-active, but their evidence base is still largely built on sparse studies. Laser-based protocols are parameter-sensitive, and other caustic approaches such as heat treatment should be regarded only as proof-of-principle models. Clinically relevant progress will require standardized protocols that combine surface chemistry, polymerization behavior, adhesive compatibility, nanoleakage, biological safety, pulpal relevance, and long-term aging. Well-designed clinical studies are especially needed to determine whether any deproteinization protocol provides a substantial, reproducible, and clinically worthwhile advantage over contemporary adhesive procedures. Until such evidence exists, dentin deproteinization remains an experimental or adjunctive concept.

## Figures and Tables

**Table 1:** Lasers used in dentin deproteinization, their parameters and corresponding references.

Laser Type	Nd:YAG	Er,Cr:YSGG	Er:YAG
Elemental composition	Yttrium, neodymium, aluminum, oxygen	Erbium, chromium, yttrium, scandium, gallium, garnet	Yttrium, erbium, aluminum, oxygen
Parameters	1064 nm Not effective for caries removal	2780 nm 100–300 mJ per pulse Water-dependent	2940 nm 100–40 mJ per pulse Water-dependent
References	[115,116]	[116]	[113,117,118]

**Table 2:** Mechanistic and translational overview of dentin deproteinization agents. The table compares oxidizing irrigants, proteolytic enzymes, and PG/GAG-targeting enzymes according to reported protocol windows, substrate targets, polymerization compatibility, bonding/durability implications, biological cautions, and clinical readiness. Concentration, pH, and application-time ranges reflect values reported in the literature and should be interpreted cautiously because protocols remain heterogeneous and are not yet standardized for routine adhesive dentistry.

Agent [Class]	Typical Protocol Window *	Main Use	Polymerization Compatibility	Bonding and Durability Interpretation	Clinical Caution and Readiness
**Sodium hypochlorite (NaOCl)** [Oxidizing irrigant]	6–10%; pH 11–13; Typically 15–120 s	Smear-layer or post-etching dentin; strongest evidence for rapid organic removal	High oxidative carry-over; thorough rinsing and antioxidant/reducing step often required	Immediate results are heterogeneous; durability may improve when organic residues are removed and residual oxidants are controlled	Most studied, but cytotoxic and oxidizing if uncontrolled; not routine without neutralization and protocol validation
**Hypochlorous acid** **(HOCl)** [Oxidizing irrigant]	0.004–0.02%; pH 6–7.5; Typically 15–60 s	Mainly smear-layer deproteinization; limited post-etching evidence	Lower chlorine residue than NaOCl, but wash-out/metal chloride or sulfinate strategies may still be needed	Potentially favorable for one-step self-etch adhesives within selected application/wash-out windows	Promising but highly protocol-sensitive; biological and long-term restorative data remain limited
**Calcium hypochlorite [Ca(OCl)_2_]** [Oxidizing irrigant]	1–15%; pH >11; Typically 15–60 s	Smear-layer or post-etching dentin; emerging alternative to NaOCl	Oxidizer; concentration, time, and rinsing must be controlled	Promising laboratory findings, but limited long-term restorative data	Emerging option; solution preparation and biological effects require standardization
**Bromelain** [Proteolytic enzyme]	6–10%; near-neutral pH; 30–60 s studied	Smear-layer and post-etching protocols	No persistent oxidizing carry-over; neutralization generally not required	Often favorable immediate outcomes, but surface-chemical kinetics are less established than for oxidizers	Promising selective alternative; potential allergenicity/formulation stability and aging evidence require clarification
**Papain** [Proteolytic enzyme]	8–10%; near-neutral pH; 30–60 s to 1 min studied	Smear-layer and post-etching protocols; purified enzyme and commercial gels differ	No persistent oxidizing carry-over; formulation-dependent	Often favorable or neutral; effect depends on concentration and formulation	Promising but formulation-dependent; commercial products should not be assumed equivalent to purified papain
**Peracetic acid (PAA)** [Oxidizing irrigant]	1–2%; pH formulation-dependent; commonly ~60 s	Mostly endodontic smear-layer/removal models	Oxygen release may inhibit free-radical polymerization	May aid smear-layer removal and sealer adhesion; restorative bonding evidence is limited	Adjunctive/endodontic relevance stronger than restorative evidence; cytotoxicity/erosion depend on formulation
**Chlorine dioxide** **(ClO_2_)** [Oxidizing irrigant]	0.0014–13.8%; pH formulation-dependent; commonly ~60 s	Mostly endodontic/disinfection models; protein removal less quantified	Oxidizing species possible; bonding effect insufficiently characterized	Evidence weaker than NaOCl/HOCl; deproteinization capacity not well quantified	Insufficient for routine restorative deproteinization; gas/oxidizer handling and cytotoxicity concerns
**Chondroitinase ABC** [PG/GAG enzyme]	0.1–0.25 U/mL; pH 7.4–8.0; Hours, not seconds	Post-etching PG/GAG targeting	No oxidizing carry-over	Mechanistically useful for probing PG/GAG contribution, but protocols are too long for chairside use	Research enzyme only; not a clinical material
**Hyaluronidase** [PG/GAG enzyme]	150–300 U/mL; pH 6.5–7.4; Clinical window not established	Exploratory PG/GAG targeting	No oxidizing carry-over	Exploratory evidence; insufficient clinical protocol data	Research enzyme only; not a clinical material
**Pepsin** [Acidic protease]	mg/mL range; pH 1.5–2.0; Long acidic exposure	Experimental post-etching proteolysis	Acidic pH incompatible with routine bonding	Mechanistically plausible but clinically impractical	Acidic protease; not a clinical bonding step
**Trypsin** [Protease and PG-protein targeting]	1–10 mg/mL; pH 7.5–8.5; Long exposure	Experimental post-etching PG/protein targeting	No oxidizing carry-over	Better documented than other PG/GAG routes, but not chairside-ready	Research enzyme; long application time prevents routine use

* Based on parameters reported in the literature.

## Data Availability

The original contributions presented in this study are included in the article. Further inquiries can be directed to the corresponding author.
